# Evaluation of Quality of Life among Dental Professionals by Using the WHOQOL-BREF Instrument in Eastern Province of Saudi Arabia

**DOI:** 10.1155/2020/5654627

**Published:** 2020-12-23

**Authors:** Nabras Alrayes, Hend Alshammary, Marwah Alamoudi, Banin Alfardan, Muhanad Alhareky, Muhammad Nazir

**Affiliations:** ^1^College of Dentistry, Imam Abdulrahman Bin Faisal University, P. O. Box 1982, Dammam 31441, Saudi Arabia; ^2^Department of Preventive Dental Sciences, College of Dentistry, Imam Abdulrahman Bin Faisal University, P. O. Box 1982, Dammam 31441, Saudi Arabia

## Abstract

**Objective:**

To evaluate the quality of life (QOL) of dental professionals in the Eastern Province of Saudi Arabia.

**Methods:**

This cross-sectional study recruited dental professionals (general dentists, specialists, and consultants) from public and private sectors in the Eastern Province of Saudi Arabia. The World Health Organization's QOL Assessment-BREF (WHOQOL-BREF) questionnaire was administered among study participants. The questionnaire addresses four domains of QOL which are physical, psychological, social relationships, and environment.

**Results:**

There were 313 dental professionals in the study with a mean age of 35.72 (8.58) years. The mean score of QOL in the sample was 63 (13.9) on a 0–100 scale. 75% of the participants rated their QOL as good or very good. Of four domains, the social relationship domain had the highest mean score of QOL (67.04 SD: 23.52) and the physical domain had the lowest score (59.66 SD: 14.69). There were significant differences in the QOL of consultants (66.46 SD: 12.55), specialists (65.42 SD: 12.91), and general dentists (61.06 SD: 14.18) (*P* 0.010). The participants with medical illness had significantly lower QOL (56.91 SD: 12.83) than those without medical illness (63.67 SD: 13.92) (*P* 0.01). There were significant differences in the QOL of participants with 0–10 years since graduation (61.97 SD: 14.71), 11–20 years (61.92 SD: 13.56), and more than 20 years (68.53 SD: 10.71) (*P* 0.009).

**Conclusion:**

The qualifications, medical illness, and years since graduation were important determinants of QOL among dental professionals. Measures should be taken to improve QOL of dental professionals which can enhance the quality of patient care.

## 1. Introduction

The quality of life (QOL) is a multidimensional construct that can be defined as “the individuals' perception of their position in life in the context of the culture and value systems in which they live, and in relation to their goals, expectations, standards, and concerns” [1]. Generally, the QOL can be determined by daily activities, such as social interaction, personal care, comfort, and mobility. In 1920, Pigou AC was the first person to introduce the concept of QOL in economics, and then, this term was introduced to the medical and dental fields after World War II [2]. Currently, different definitions and explanations of QOL exist in different healthcare professions [3]. High quality dental care and patient satisfaction require physical and mental efforts of the dentist, and this makes QOL a vital concept in today's dental practice [4].

The healthcare providers are susceptible to occupational risks that can affect their quality of work through exposure to chemicals, radiations, physical, and psychosocial hazards [5]. Therefore, the World Health Organization (WHO) selected healthcare providers as a priority group for the improvement in their workplace health and safety in the work plan 2009–2012 (priority 1.4) [6]. In dental practice, the physical and mental stresses negatively influence dentists' QOL and their general health [4, 7]. It is known that job-related stresses are associated with certain systemic diseases, such as coronary heart disease and musculoskeletal disorders [7]. These stresses can reduce dentists' productivity and clinical performance as well as their satisfaction levels. Moreover, their social life including relationships with their families can be affected [4].

The literature shows studies of QOL among healthcare professionals. A study of physicians working in the emergency department showed that male physicians had a better QOL than female physicians [8]. A recent study of the intensive care professionals revealed that the physically active participants had a higher quality of life than the physically inactive professionals [9]. Similarly, half of the medical students were shown to have low QOL in psychological and social domains, and female medical students demonstrated lower QOL compared to male students [10]. In the dental profession, a study among dentists in South Canara, India, found that the majority of dentists rated their QOL as very good [11]. Another study observed better QOL in married dentists compared with single dentists in the United Arab Emirates [4]. Recently, dentists were shown to demonstrate high health-related quality of life in Iran [12]. However, limited data regarding the QOL of dentists underscore the importance of further investigation. Therefore, the aim of this study was to evaluate the QOL of male and female dentists in the Eastern Province of Saudi Arabia.

## 2. Materials and Methods

### 2.1. Study Population

A cross-sectional study recruited both female and male dentists from public and private sectors in Khobar, Dammam, Dhahran, Jubail, Alhasa, and Qatif cities in the Eastern Province of Saudi Arabia. The team of researchers visited clinics and hospitals in these cities, and dentists were contacted in person. Maximum three visits were performed if dentists were unable to provide their responses in the first or second visits. This helped achieve an adequate response rate for the study. The hard copies of the questionnaire were delivered to dentists in their dental offices. The selection of dentists was performed using a convenience sampling technique. The dentists willing to participate in the study were requested to fill out the questionnaire according to what they felt during the last 2 weeks [13]. A sample of 377 dentists was calculated based on a 95% confidence level, 5% margin of error, 50% response distribution, and an approximate dentist population in the Eastern Province (*n* = 2000).

### 2.2. Measurement Instrument

The main objective of this study was to evaluate the QOL of dentists. To attain this goal, a World Health Organization' QOL Assessment-BREF (WHOQOL-BREF) questionnaire was used. The questionnaire addresses four domains of QOL which are physical, psychological, social relationships, and environmental areas. The physical domain includes pain and discomfort facet, and it assesses the physical condition of a person and how it interferes with his/her daily life activities. It also includes sleep, rest, energy, and fatigue facets that evaluate sleep and rest of the person and the energy and the willingness of a person to perform everyday tasks.

The psychological domain examines how often a person experiences positive and negative perceptions and what impact they have on a person's daily functioning. Furthermore, it assesses people's way of thinking, how capable they are of making decisions, and the way people evaluate their worth/self-esteem. Body image and appearance facet explore the way people think of their external appearance and to what extent they are satisfied with it. The social domain addresses the personal relationships and social support a person has and their impact on his/her life. It also examines if the person is achieving the love and support, he/she desires from these intimate relationships, family, and friends.

The environmental domain includes physical safety facet which examines if a person feels secure from any physical harms. It also includes home environment facet and financial resources facet that explores to what extent a person is satisfied with his/her income and whether it is enough to meet the needs of a decent life. In addition, other facets in this domain assess various aspects of life such as the quality and availability of nearby healthcare services, opportunities for acquiring new information and skills, persons' capacity to enjoy their leisure time, physical environment, and availability of transport services. The WHOQOL-BREF questionnaire is a valid instrument, and it demonstrates good internal consistency [13].

#### 2.2.1. Pretesting of the Questionnaire

The WHOQOL-BREF questionnaire, English version, was pretested among a small sample of 22 dentists after taking permission from the administrators of the dental clinics in Khobar, Jubail, and Qatif cities. Based on the feedback obtained in the pretesting and discussions among researchers, only “living as married” option was removed from the marital status question in the questionnaire because it is against the cultural norms in Saudi Arabia. Pretesting also helped evaluate estimated time to fill the questionnaire, responses of the respondents, the relevance of questions, and any problems associated with the formulation of questions.

#### 2.2.2. Scoring System Used

The scoring system for the WHOQOL-BREF is performed for four domains. The scoring of the domains is done in a positive direction. The higher the positive facets score, the higher the quality of life. The sum of each domain item indicates the domain's final score. The sum is then multiplied by 4 to make the score of the domain compatible with the WHOQOL-100 score. Then, the sum of all the domains is converted into 0–100 scale by the following formulas [13].

(1) $$\begin{array}{c} \text{Transformed score}=\left(\text{score}-4\right)\times \left(\frac{100}{16}\right),\\ \text{Physical domain}=\left(\left(6-Q3\right)+\left(6-Q4\right)+Q10+Q15+Q16+Q17+Q18\right)\times 4,\\ \text{Psychological domain}=\left(Q5+Q6+Q7+Q11+Q19+\left(6-Q26\right)\right)\times 4,\\ \text{Social relationships domain}=\left(Q20+Q21+Q22\right)\times 4,\\ \text{Environment domain}=\left(Q8+Q9+Q12+Q13+Q14+Q23+Q24+Q25\right)\times 4. \end{array}$$

The study was approved by the ethics committee at the College of Dentistry at Imam Abdulrahman Bin Faisal University. The permission was obtained from the medical director or the general manager of the hospitals/clinics to distribute the surveys. The dentists were informed about the aim and objectives of the study, and their written informed consents were obtained. The use of anonymous questionnaire ensured the confidentiality and privacy of the respondents.

### 2.3. Statistical Analysis

SPSS IBM version 22 (IBM SPSS Statistics for Windows, Version 22.0, IBM Corp: Armonk, NY) was used for statistical analyses. Descriptive statistics included means, standard deviations, frequencies, and percentages. Independent *t*-tests and one-way ANOVA tests were performed after fulfilling the normality assumption of QOL variable using the Shapiro–Wilk test. Independent *t*-tests were performed to compare the mean score of QOL between male and female dentists, private and public dentists, married and single/divorce dentists, and dentists with and without medical illness. The one-way ANOVA test was performed to compare the QOL score in three categories of dentists regarding monthly income, years of graduation, and qualifications. A *P* value of less than 0.05 was considered statistically significant.

## 3. Results

Out of 377 dentists, 313 returned completed questionnaires giving a response rate of 83%. The study included 56.2% of males and 43.8% of females with a mean age of 35.72 (8.58) years. Slightly more than half of the participants (52.1%) had 0–10 years of experience since graduation and were general dentists (55.6%). Medical illness was reported by 9.9% of the sample. The majority of participants (71.6) were married and were from private practice (69.3%) (Table 1).

Table 2 shows the mean score of QOL in four domains of the instrument. The mean QOL score among participants was 63 (13.9) on a 0–100 scale. Of four domains, the social relationship domain had the highest mean score of QOL (67.04 SD: 23.52) and the physical domain demonstrated the lowest score (59.66 SD: 14.69).

Table 3 presents the comparison of mean scores of each domain in different categories of participants. The mean score for the physical domain significantly differed among general dentists, specialists, and consultants (*P*=0.049). Similarly, general dentists, specialists, and consultants showed significant differences in the mean score of the psychological domain (*P*=0.003). In the social relationships domain, consultants demonstrated the highest mean score (*P* 0.016). The participants without medical illness showed higher mean scores in the psychological domain (*P*=0.035), social relationships domain (*P*=0.012), and environment domain (*P*=0.046) than those with medical illness (Table 3).

Results of comparison of the mean score of QOL in different categories of participants are shown in Table 4. There were significant differences in the QOL of consultants (66.46 SD: 12.55), specialists (65.42 SD: 12.91), and general dentists (61.06 SD: 14.18) (*P* 0.010). Similarly, QOL was significantly associated with years since graduation (*P* 0.009). The participants without medical illness had significantly higher QOL (63.67 SD: 13.92) than those with medical illness (56.91 SD: 12.83) (*P* 0.01). There were significant differences in the QOL of participants with 0–10 years since graduation (61.97 SD: 14.71), participants with 11–20 years since graduation (61.92 SD: 13.56), and those with more than 20 years since graduation (68.53 SD: 10.71) (*P* 0.009) (Table 4).

**Figure 1:**
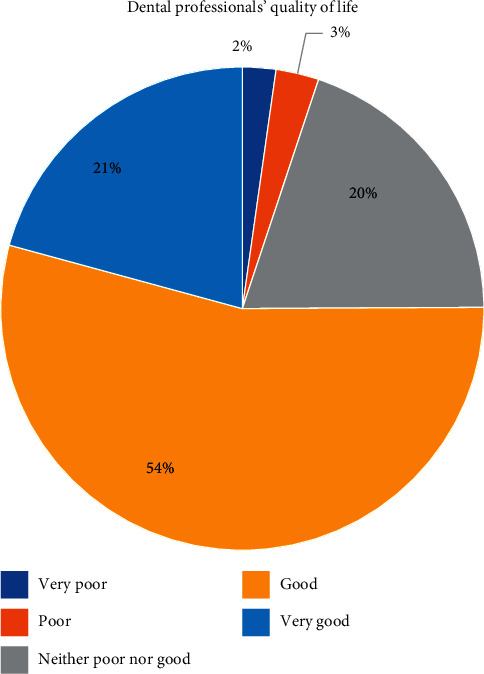
Distribution of dental professionals' perception about their QOL.

Distribution of dental professionals' perception about their QOL is shown in Figure 1. 75% of participants considered their QOL good or very good and 5% thought it poor or very poor.

## 4. Discussion

Our study assessed the QOL of dental professionals and its influencing factors in Saudi Arabia. The majority of dental professionals (75%) in our study indicated that they had a good or very good QOL. The study demonstrated the highest mean score in the social relationships domain. Similar studies by Abraham et al. [4] and Doshi et al. [11] have shown the highest mean score in the social relationships domain. Personal relationships and social support are the components assessed in the social relationships domain which could be influenced by the qualifications of dental professionals [4].

According to our data, the highest QOL was observed in consultants and the lowest in general dentists. This finding is consistent with the results of a study of dental professionals in the United Arab Emirates [4]. High QOL in consultants/specialists can be attributed to different factors such as financial stability, security, social and healthcare, better chances of obtaining new information and skills, and opportunities for leisure activities [4]. Moreover, higher job satisfaction and greater income in specialists than general practitioners are possible contributing factors for improved QOL in specialists [14, 15]. Additionally, having a postgraduate degree may result in high self-esteem and positive feelings [11]. Our study results also showed higher mean scores in all four domains for consultants/specialists than general dentists.

Gender differences exist among dentists regarding workplace practices and career satisfaction. It is known that more male dentists own dental practices, whereas more female dentists work as associates, and male dentists are more satisfied with their career than female dentists [15]. Interestingly, no significant differences were observed between male and female dental professionals in the present study. Similarly, dentists in the United Arab Emirates [4] and Iran [12] demonstrated no significant differences in QOL with regard to gender. This shows that both male and female dental professionals are exposed to somewhat similar physical, psychological, social, and environmental influences that affect their QOL. However, a previous study of physicians demonstrated higher QOL in males compared with females [8].

Dental professionals working in public sector receive monthly salaries from the government and enjoy a permanent and secure job with benefits of getting higher education/specialists training and retirement plans in Saudi Arabia. On the other hand, patients pay for dental services to dental professionals in the private sector who can earn more money and enjoy greater comfortability and autonomy over the public sector dental professionals. The dentists in the private sector own the business of dental practice and can take time for vacations and attendance of conferences and seminars [16]. In the private sector, dentists can work in a solo practice or in a large group practice, and the dentists in large group practice are known to have increased job satisfaction with income and benefits [17]. Therefore, in the context of the employment sector, dental professionals both in public and private sectors enjoy QOL in their practice settings. This was depicted in the present study as there was no significant difference in QOL among dental professionals in private and public jobs. In a recent study from a Middle Eastern country, the dentists in public and private sectors also documented no significant differences in the overall job satisfaction [16].

Healthcare providers become more competent and get attached to their job with more years of experience [18]. The quality of life of dentists may improve with age because experienced dentists have established relationships in their work community, and they are more able to balance between their personal life demands and their career [4]. Similar trends were observed in the present study where the years since graduation have a significant influence on the QOL of dental professionals and the participants with more than 20 years since graduation demonstrated the highest score of QOL.

The physical domain, in our study, has the lowest mean score which is contrary to what was reported by Nunes et al. who indicated the highest mean score (70.3; SD = 14.6) for the physical domain [19]. Likewise, another study by Salehi Zeinabadi et al. found that the physical domain had the highest mean score among all domains of QOL [12]. Inadequate ergonomic practices such as long working hours and high physical demands required to achieve patients' wellbeing predispose dentists to work-related musculoskeletal disorders [20, 21]. In addition, dentists are at an increased risk of burnout due to high job strain which can develop early in their professional career [22]. Researchers showed that work-related musculoskeletal disorders are common in dental practitioners in Riyadh and Jeddah, Saudi Arabia [20, 21]. The high occurrence of work-related musculoskeletal disorders in dentists in the country may account for the lowest mean score in the physical domain in our study.

In the present study, medically ill participants had a significantly lower QOL compared with their medically fit counterparts. This finding is in agreement with the results of a study by Doshi et al. [11]. Our study further confirmed significantly lower mean scores in psychological, social relationships, and environment domains. A recent study in the Eastern Province of Saudi Arabia revealed that patients with diabetes mellitus had impaired QOL mostly in the terms of pain/discomfort and mobility, and improved QOL was found in patients without diabetic complications [23]. Another study found that hypertensive patients are linked to a low QOL especially in the psychological domain [24]. These studies can explain why medically ill participants may have lower QOL, taking into consideration that diabetes mellitus and hypertension are highly prevalent in Saudi Arabia [25, 26].

There are certain limitations to the study. The study used a convenience sample of dentists from the Eastern Province; hence, caution should be exercised when generalizing the study results to all dental professionals in Saudi Arabia. Furthermore, the cross-sectional study design is limited in casual inference between assessed factors and QOL. Given the importance of quality of life in the context of dental practice and the lack of knowledge base on this topic, it is suggested that a multicenter study should be conducted in different countries. The study can evaluate the impact of the presence or absence of health policies aimed at improving QOL of dentists.

## 5. Conclusion

The study demonstrated good QOL in the majority of dental professionals. The consultants exhibited the highest QOL compared with specialists and general dentists. Experienced dental professionals with more than 20 years since graduation reported the highest QOL. Low QOL was observed among dental professionals with a medical problem. Dental professionals demonstrated the lowest QOL score in the physical domain that encompasses physical conditions and daily life activities. Decision-makers in healthcare should develop and implement policies and programs to improve the quality of life of dental professionals in the country. General practitioners, dental professionals with less experience, and those with medical problems should be given priority during the development of QOL improvement initiatives.

## Figures and Tables

**Table 1:** Distribution of study variables among dental professionals.

Study variables	*N* (%), *N* = 313
Gender	
Male	176 (56.2)
Female	137 (43.8)

Marital status	
Married	224 (71.6)
Single/divorce	89 (28.4)

Year of graduation	
0–10 years	163 (52.1)
11–20 years	100 (31.9)
>20 years	50 (16)

Type of job	
Government job	96 (30.7)
Private job	217 (69.3)

Qualification	
General dentists	174 (55.6)
Specialists	94 (30)
Consultants	45 (14.4)

Basic dental qualification	
Government college	201 (64.2)
Private college	112 (35.8)

Average monthly income	
Less than 10,000 SAR	88 (28.1)
10,000–20,000 SAR	110 (35.1)
More than 20,000 SAR	115 (36.7)

Medical illness	
Yes	31 (9.9)
No	282 (90.1)

**Table 2:** Four domains of quality of life among dentists.

QOL domains	Mean (SD) (0–100)
Physical domain	59.66 (14.69)
Psychological domain	62.59 (16.07)
Social relationships domain	67.04 (23.52)
Environment domain	62.61 (17.52)
Overall score	63 (13.9)

**Table 3:** Association between sociodemographic factors and different domains of QOL in dentists.

Study variables	Physical domain, mean (SD)	*P* value	Psychological domain, mean (SD)	*P* value	Social relationships domain (SD)	*P* value	Environment domain (SD)	*P* value
Gender								
Male	59.31 **(**15.31)	0.632	63.23 **(**15.78)	0.425	68.24 **(**22.44)	0.31	63.41 **(**16.99)	0.363
Female	60.11 **(**13.91)		61.76 **(**16.46)		65.51 **(**24.83)		61.59 **(**18.19)	

Marital status								
Married	58.88 **(**14.05)	0.136	61.65 **(**15.47)	0.103	66.24 **(**23.61)	0.335	61.75 **(**16.8)	0.167
Single/divorce	61.63 **(**16.13)		64.94 **(**17.36)		69.09 **(**23.31)		64.78 **(**19.13)	

Year of graduation								
0–10 years	59.24 **(**15.02)	0.507	60.59 **(**16.28)	0.002^*∗*^	65.39 **(**25.11)	0.071	62.67 **(**17.61)	0.014^*∗*^
11–20 years	59.24 **(**15.2)		62.25 **(**15.8)		66.27 **(**22.25)		59.59 **(**18.17)	
>20 years	61.9 **(**12.53)		69.75 **(**14.07)		74.01 **(**16.89)		68.44 **(**14.41)	

Type of job								

Government job	57.38 **(**12.91)	0.068	63.27 **(**16.01)	0.619	66.59 **(**23.06)	0.818	63.48 **(**18.36)	0.561
Private job	60.67 **(**15.35)		62.29 **(**16.1)		67.25 **(**23.77)		62.23 **(**17.16)	

Qualification								
General dentists	60.01 **(**15.03)	0.049^*∗*^	59.8 **(**16.73)	0.003^*∗*^	63.65 **(**24.62)	0.016^*∗*^	59.61 **(**17.71)	0.002^*∗*^
Specialists	57.15 **(**15.15)		66.1 **(**14.53)		71.28 **(**22.52)		67.05 **(**16.32)	
Consultants	63.57 **(**11.38)		66.01 **(**14.66)		71.33 **(**19.1)		64.93 **(**17.23)	

Basic dental qualification from:								
Government college	59.22 **(**14.57)	0.479	63.02 **(**16.14)	0.525	66.17 **(**22.8)	0.38	62.69 **(**16.62)	0.918
Private college	60.45 **(**14.97)		61.81 **(**15.98)		68.62 **(**24.79)		62.47 (19.1)	

Average monthly income								
Less than 10,000 SAR	60.43 **(**15.15)	0.545	61.12 **(**16.67)	0.454	66.96 **(**21.99)	0.951	61.11 **(**16.98)	0.55
10,000–20,000 SAR	58.42 **(**15.23)		62.33 **(**13.96)		67.59 **(**24.5)		62.53 **(**15.54)	
More than 20,000 SAR	60.26 **(**13.85)		63.95 **(**17.46)		66.6 **(**23.88)		63.83 **(**19.63)	

Medical illness								
Yes	57.18 **(**13.72)	0.322	56.81 **(**16.17)	0.035^*∗*^	56.99 **(**25.46)	0.012^*∗*^	56.65 **(**14.58)	0.046^*∗*^
No	59.93 **(**14.8)		63.22 **(**15.96)		68.15 **(**23.07)		63.26 **(**17.71)	

^*∗*^Statistically significant.

**Table 4:** Association between sociodemographic factors and QOL in dentists.

Study variables	QOL, mean score (SD)	*P* value
Gender		
Male	63.59 (13.76)	0.396
Female	62.25 (14.19)	
Marital status		
Married	62.17 (13.38)	0.092
Single/divorce	65.11 (15.14)	
Year of graduation		
0–10 years	61.97 (14.71)	0.009^*∗*^
11–20 years	61.92 (13.56)	
>20 years	68.53 (10.71)	
Type of job		
Government job	62.77 (13.9)	0.841
Private job	63.11 (14)	
Qualification		
General dentists	60.82 (14.5)	0.007^*∗*^
Specialists	65.4 (12.91)	
Consultants	66.46 (12.55)	
Basic dental qualification from:		
Government college	62.82 (13.38)	0.752
Private college	63.34 (14.97)	
Average monthly income		
Less than 10,000 SAR	62.41 (13.43)	0.772
10,000–20,000 SAR	62.72 (13.69)	
More than 20,000 SAR	63.73 (14.65)	
Medical illness		
Yes	56.91 (12.83)	0.010^*∗*^
No	63.67 (13.92)	

^*∗*^Statistically significant.

## Data Availability

The data used to support the findings of this study are available from the corresponding author upon request.

## References

[1] The WHOQOL Group (1998). Development of the World Health Organization WHOQOL-BREF quality of life assessment. The WHOQOL group. *Psychological Medicine*.

[2] Baiju R.M., Peter E., Varghese N. O., Sivaram R. (2017). Oral health and quality of life: current concepts. *Journal of Clinical and Diagnostic Research: JCDR*.

[3] Haraldstad K., Wahl A., Andenæs R. (2019). A systematic review of quality of life research in medicine and health sciences. *Quality of Life Research*.

[4] Abraham S. B., Amini A. M. A., Khorshed N. E., Awad M. (2018). Quality of life of dentists. *European Journal of Dentistry*.

[5] Senthil A., Anandh B., Jayachandran P. (2015). Perception and prevalence of work-related health hazards among health care workers in public health facilities in southern India. *International Journal of Occupational and Environmental Health*.

[6] World Health Organization (2017). *Global Network of WHO Collaborating Centres for Occupational Health*.

[7] Alexopoulos E. C., Stathi I.-C., Charizani F. (2004). Prevalence of musculoskeletal disorders in dentists. *BMC Musculoskeletal Disorders*.

[8] Fernandez-Prada M., González-Cabrera J., Torres F. G., Iribar-Ibabe C., Peinado J. M. (2014). Gender influence on health related quality of life among resident physicians working in an emergency department. *Revista medica de Chile*.

[9] Freire C. B., Dias R. F., Schwingel P. A. (2015). Quality of life and physical activity in intensive care professionals from middle Sao Francisco. *Revista Brasileira de Enfermagem*.

[10] Pagnin D., de Queiroz V. (2015). Comparison of quality of life between medical students and young general populations. *Education for Health*.

[11] Doshi D., Vinaya K., Jain A., Kotian S. (2011). Quality of life among dentists in teaching hospitals in South Canara, India. *Indian Journal of Dental Research*.

[12] Zeinabadi M. S., Safaian G., Mirmohammadkhani O., Mirmohammadkhani M., Ameli N. (2018). Evaluation of health-related quality of life among dentists in Semnan, Iran, 2015-2016. *Middle East Journal of Rehabilitation and Health*.

[13] World Health Organization (1998). *Programme on Mental Health: WHOQOL User Manual*.

[14] Azevedo W. F. d., Mathias L. A. d. S. T. (2017). Work addiction and quality of life: a study with physicians. *Einstein (São Paulo)*.

[15] Ayers K. M. S., Thomson W. M., Rich A. M., Newton J. T. (2008). Gender differences in dentists’ working practices and job satisfaction. *Journal of Dentistry*.

[16] Al-Buainain F. S., Alzarouni A. A., Alshamsi H. A., Arab A. H., Bader F., Awad M. (2019). Job satisfaction of U.A.E. Dental practitioners. *European Journal of Dentistry*.

[17] Lo Sasso A. T., Starkel R. L., Warren M. N., Guay A. H., Vujicic M. (2015). Practice settings and dentists’ job satisfaction. *The Journal of the American Dental Association*.

[18] Taycan O., Erdoğan Taycan S., Çelik C. (2013). The impact of compulsory health service on physicians and burnout in a province in Eastern Anatolia. *Turk Psikiyatri Dergisi*.

[19] Nunes M. D. F., Freire M. D. C. M. (2006). Qualidade de vida de cirurgiões-dentistas que atuam em um serviço público. *Revista de Saúde Pública*.

[20] Meisha D. E., Alsharqawi N. S., Samarah A. A., Al-Ghamdi M. Y. (2019). Prevalence of work-related musculoskeletal disorders and ergonomic practice among dentists in Jeddah, Saudi Arabia. *Clinical, Cosmetic and Investigational Dentistry*.

[21] Al-Mohrej O. A., AlShaalan N. S., Al-Bani W. M., Masuadi E. M., Almodaimegh H. S. (2016). Prevalence of musculoskeletal pain of the neck, upper extremities and lower back among dental practitioners working in Riyadh, Saudi Arabia: a cross-sectional study. *BMJ Open*.

[22] Singh P., Aulak D. S., Mangat S. S., Aulak M. S. (2016). Systematic review: factors contributing to burnout in dentistry. *Occupational Medicine*.

[23] Alshayban D., Joseph R. (2020). Health-related quality of life among patients with type 2 diabetes mellitus in Eastern Province, Saudi Arabia: a cross-sectional study. *PLoS One*.

[24] Xiao M., Zhang F., Xiao N., Bu X., Tang X., Long Q. (2019). Health-related quality of life of hypertension patients: a population-based cross-sectional study in chongqing, China. *International Journal of Environmental Research and Public Health*.

[25] Alotaibi A., Perry L., Gholizadeh L., Al-Ganmi A. (2017). Incidence and prevalence rates of diabetes mellitus in Saudi Arabia: an overview. *Journal of Epidemiology and Global Health*.

[26] El Bcheraoui C., Memish Z. A., Tuffaha M. (2014). Hypertension and its associated risk factors in the kingdom of Saudi Arabia, 2013: a national survey. *International Journal of Hypertension*.

